# China’s colorectal cancer burden and dietary risk factors: a temporal analysis (1990–2021)

**DOI:** 10.3389/fnut.2025.1590117

**Published:** 2025-07-18

**Authors:** Qin Sun, Dengjun Bi, Yueshan Pang, Jiebin Xie

**Affiliations:** ^1^Department of Gastrointestinal Surgery, Affiliated Hospital of North Sichuan Medical College, Nanchong, Sichuan, China; ^2^North Sichuan Medical College, Nanchong, Sichuan, China; ^3^Department of General Practice, Beijing Anzhen Nanchong Hospital of Capital Medical University & Nanchong Central Hospital, Nanchong, Sichuan, China

**Keywords:** colorectal cancer (CRC), global burden of disease (GBD), dietary risk factors, colon cancer, rectal cancer

## Abstract

**Background:**

Colorectal cancer (CRC) is a major global and Chinese public health issue, and dietary factors are controllable risk factors. However, China’s CRC epidemiological trends and diet-attributable analyses based on the latest global burden of disease (GBD) data remain incomplete. This study systematically assessed China’s CRC disease burden (1990–2021) and temporal trends in diet-related risk factors via GBD 2021 data to inform precision prevention.

**Methods:**

GBD 2021 data were used to analyze age/sex differences in the incidence, prevalence, mortality, disability-adjusted life years (DALYs) and diet-related risk factors for CRC. Temporal trends in CRC burden were evaluated via joinpoint regression analysis. To enable comparisons across populations with differing age structures, we calculated age-standardized rates (ASRs) via the GBD world standard population. The estimated annual percentage change (EAPC) trends were assessed.

**Results:**

In 2021, China had 658321.36 new CRC cases (315.63% increase vs. 1990) and 275129.23 deaths (130.61% increase), with males showing greater burden increases. The 15–49-year-old had the fastest incidence growth (EAPC = 3.40%); those ≥75 years of age had the highest incidence (255.34/100000 people). Among the dietary risk factors, low milk intake caused the most deaths in 2021 (51030.32 cases), followed by low whole grain intake (49990.79 cases). From 1990 to 2021, processed meat-related deaths rose the most (274.71%), with higher proportions of young adults; low-calcium diet deaths significantly declined (EAPC = -0.48%). Females had the highest share of low-milk deaths, whereas males had the most low-whole-grain deaths.

**Conclusion:**

China’s CRC burden is increasing overall, with increasing sex differences and coexisting youth and later life trends, driven by dietary shifts. Urgent promotion of increased milk and whole-grain intake, alongside reduced processed meat consumption, is needed, with tailored strategies for different populations.

## Introduction

As a major global health concern, colorectal cancer (CRC) is the second leading cause of cancer-related mortality globally (1). In 2019, 607,900 new CRC cases were recorded in China—a 474.0% incidence increase (30.6/100,000) (2). Since 2020, CRC has ranked among the top five most prevalent cancers in China (3). The incidence in males is nearly double that in females (2). Key CRC risk drivers include unhealthy diets (4, 5), environmental chemical exposure (6), gut microbiota dysbiosis (7), and prolonged antibiotic use (8). Diet has received increasing attention as a key CRC risk factor. In 2019, China’s diet-attributable CRC mortality rate reached 90.41/100,000, increasing annually by 2.05% since 1990 (9). These findings underscore the substantial CRC burden in China, necessitating urgent identification of modifiable dietary risk factors and implementation of targeted prevention strategies to mitigate this growing public health challenge.

Dietary factors play a pivotal role in CRC pathogenesis, with approximately 50% of colon cancer cases being preventable through dietary modifications (10). Protective dietary patterns characterized by increased fiber, calcium, and yogurt intake, coupled with reduced alcohol and red meat consumption, are associated with decreased CRC risk (11). Global analyses by Liang et al. (12) reported 365,752 diet-attributable CRC deaths in 2019, constituting one-third of total CRC mortality. Liu et al. (9) identified six dietary risk factors for CRC in China: low fiber, inadequate milk/calcium/whole grains, and excessive red/processed meat. Low milk intake was associated with the highest attributable mortality (3.71/100,000) (9). While the study by Liu et al. (9), which was based on GBD 2019 data, did not characterize the temporal trends for each individual diet-related risk factor in CRC or conduct age-stratified analyses, our investigation, which utilized the more recent GBD 2021 dataset, prioritizes the examination of both temporal changes in the CRC burden attributable to each specific dietary risk factor and its age-specific stratification. These results are beneficial for formulating targeted prevention and control policies, reducing the CRC disease burden in China, and promoting healthy outcomes for Chinese citizens.

The GBD 2021 study systematically collates and analyzes multinational epidemiological data across diseases and injuries. Leveraging this robust database, our study investigated temporal trends in CRC burden and diet-attributable CRC in China from 1990 to 2021. We performed sex- and age-stratified analyses of incidence, prevalence, mortality and disability-adjusted life years (DALYs), providing updated insights into 31-year epidemiological patterns. This research aims to elucidate China’s evolving CRC landscape, emphasizing actionable dietary risk factors. The results of this study can help draw the attention and focus of individuals, food-related companies, and health policymakers. They also serve as a reference for Chinese policymakers to formulate relevant policies, thereby reducing the disease burden of CRC.

## Methods

### Data acquisition and sources

This study utilized data from the GBD 2021 dataset, which comprehensively evaluates the health impacts of 371 diseases and injuries and 88 risk factors across 21 regions and 204 countries/territories from 1990 to 2021 (13). The cases and age-standardized rates (ASRs) (per 100,000 people) of CRC incidence, prevalence, mortality, disability-adjusted life years (DALYs), and diet-related risk factors (red meat intake, processed meat intake, low calcium intake, low fiber intake, low milk intake, low whole grain intake) for China were extracted from the GBD 2021 dataset via the GBD Results Tool^1^. The GBD synthesizes data from sources such as household and health examination surveys and censuses, ground-sensing or remote-sensing data, and administrative records, which are standardized and modeled for global comparability (13, 14). The study involved secondary analysis of anonymized aggregate data, with no access to personally identifiable information.

### Exposure definition

GBD 2021 employs the comparative risk assessment framework to estimate risk factors, explicitly addressing overlapping risks and confounding (14). GBD 2021 refined the specification of the mediation matrices and re-evaluated the theoretical minimum risk exposure levels, summary exposure values, and other parameters through meta-regression or alternative methodological approaches (13, 14). Furthermore, the framework leverages mediation matrices to quantify the relationship between CRC and diet-related risk factors (14). GBD 2021 identified six diet-related CRC risk factors: inadequate milk intake (recommended intake (RI): 360–500 g/day), low whole grain consumption (RI: 140–160 g/day), insufficient dietary calcium (RI: 1.06–1.10 g/day), high red/processed meat intake (any consumption), and inadequate dietary fiber (RI: 21–22 g/day) (14).

### Temporal trend analysis

Temporal trends in CRC burden were evaluated via joinpoint regression analysis via the “Segment” and “broom” R packages. The algorithm fits segmented log-linear models: log(rate) = *β*₀ + β₁·Year + Σδ_k_(Year-τ_k_)+, with τ_k_ denoting joinpoints identified through permutation testing (*α* = 0.05, max = 3 joinpoints). The estimated annual percentage change (EAPC) was calculated by fitting a log-linear regression model to age-standardized rates via the equation log(ASR_t) = *β*₀ + β₁ × t + *ε*, where ASR_t represents the age-standardized rate at time t, *β*₁ is the regression coefficient for time, and ε is the error term. The EAPC was derived as 100 × (e^β₁ – 1). The 95% confidence interval (CI) was computed via the standard error of β₁ with the following formula: 100 × (e^(β₁ ± 1.96 × SE(β₁)) – 1). Stratified analyses were conducted by sex and three age groups (15–49, 50–74, and 75+ years).

### Risk factor analysis

Risk factors contributing to the burden of CRC were analyzed via data from the GBD 2021 study. A comprehensive analysis was conducted to assess the attributable deaths or DALYs for each risk factor. The `ggplot2` R package was used for risk factor analysis to display the contributions of various risk factors to the overall disease burden.

### Statistical analysis

All analyses and visualizations were conducted via R (version 4.2.3) and JD_GBDR software (v2.24; Jingding Medical Technology Co., Ltd.), ensuring full reproducibility through archived code and standardized GBD protocols. Descriptive statistics for key variables are reported as the means with 95% uncertainty intervals (UIs) or CIs. Statistical significance was determined when the 95% confidence interval excluded zero (*p* < 0.05).

## Results

### Increasing epidemiological trends of CRC in China

**Figure 1:**
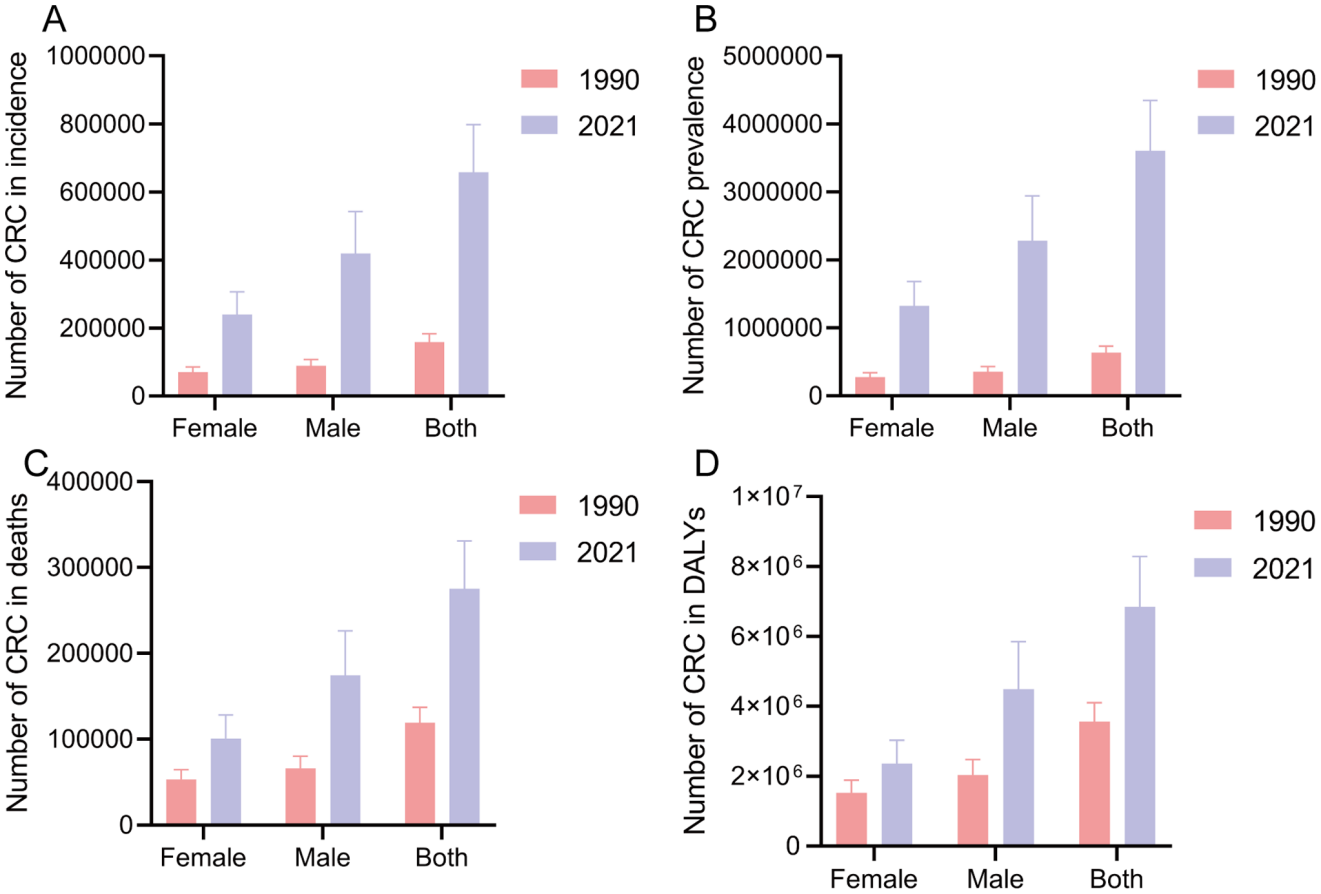
The case of incidence **(A)**, prevalence **(B)**, death **(C)**, and DALY **(D)** globally in 1990 and 2021. CRC, colorectal cancer; DALY, disability-adjusted life years.

In 1990, the total number of new CRC cases in China was 158,389.30 (95% UI: 135,418.51, 182,577.30), which increased to 658,321.36 cases (95% UI: 531,995.02, 798,063.00) by 2021. The number of new cases increased by 315.63% (95% UI: 212.40, 437.71), and the EAPC of the incidence rate increased by 4.26% (95% UI: 4.09, 4.43) (Figure 1A and Table 1). Regardless of the year, the incidence rate of CRC in males was greater than that in females (Figure 1A and Table 1). From 1990 to 2021, the number of new CRC cases in China increased significantly, especially in males, with an increase of 374.16% (95% UI: 228.68, 578.10) and an increase in the incidence rate of EAPC of 4.89% (95% CI: 4.70, 5.07) (Figure 1A and Table 1). In 2021, the number of prevalent CRC cases increased to 3,605,686.39 (95% UI: 2912080.79, 4349689.06). From 1990 to 2021, the number of prevalent cases increased by 467.28% (95% UI: 334.76, 627.13), with an increase in the EAPC of 5.46% (95% CI: 5.29, 5.63). The increase in prevalence was most significant in males, with an increase in the EAPC of 6.05% (95% CI: 5.84, 6.26) (Figure 1B and Supplementary Table 1). In 2021, the total number of deaths due to CRC was 275129.23 (95% UI: 223378.58, 330960.39). From 1990 to 2021, the number of deaths increased by 130.61% (95% UI: 73.52, 195.56), with an EAPC increase of 2.09% (95% CI: 1.99, 2.19). The EAPC increase was particularly significant in males, at 2.70% (95% CI: 2.61, 2.79) (Figure 1C and Supplementary Table 2). In 2021, the total number of DALYs due to CRC in China was 6848389.89 years (95% UI: 5,513,406.57, 8,284,228.27). From 1990 to 2021, DALYs increased by 92.09% (95% UI: 43.73, 149.56), with an EAPC increase of 1.48% (95% UI: 1.37, 1.59). The EAPC increase was particularly significant in males, at 2.10% (95% CI: 2.00, 2.21) (Figure 1D and Supplementary Table 3).

**Table 1:** Incidence of colorectal cancer in China between 1990 and 2021.

Age(y)	Gender	1990 (95% UI)		2021 (95% UI)		Case percent change (%) (95% UI)	EAPC (%) (95% CI)
		Case	ASRs	Case	ASRs		
All	Both	158389.30(135418.51,182577.30)	13.46(11.51,15.52)	658321.36(531995.02,798063.00)	46.27(37.39,56.09)	315.63(212.40,437.71)	4.26(4.09,4.43)
	Male	88370.13(69951.21,107467.33)	14.56(11.53,17.71)	419012.49(319825.35,541668.40)	57.55(43.93,74.39)	374.16(228.68,578.10)	4.89(4.70,5.07)
	Female	70019.17(55492.60,85556.58)	12.29(9.74,15.02)	239308.87(181750.35,305839.16)	34.45(26.16,44.03)	241.78(141.57,385.57)	3.35(3.17,3.53)
15–49	Both	35027.26(29409.35,40729.02)	5.25(4.41,6.11)	78692.27(62703.11,96467.59)	11.86(9.45,14.54)	124.66(68.14,196.51)	2.67(2.43,2.91)
	Male	20797.33(15728.19,25356.53)	6.03(4.56,7.36)	55925.55(41917.22,72753.16)	16.23(12.16,21.11)	168.91(88.60,281.55)	3.40(3.17,3.63)
	Female	14229.93(11034.14,18060.28)	4.42(3.42,5.61)	22766.72(16569.47,30498.39)	7.14(5.20,9.57)	59.99(6.42,141.17)	1.35(1.11,1.58)
50–74	Both	96835.84(82396.02,112017.54)	56.13(47.76,64.93)	411083.08(327662.95,500243.61)	94.76(75.53,115.31)	324.52(216.30,462.43)	1.72(1.59,1.85)
	Male	54678.60(43696.71,66993.35)	61.91(49.47,75.85)	262928.84(197241.57,344477.29)	121.59(91.21,159.30)	380.86(225.37,599.75)	2.37(2.23,2.51)
	Female	42157.24(33635.43,51701.20)	50.07(39.95,61.41)	148154.24(112496.90,191134.64)	68.10(51.71,87.85)	251.43(146.74,401.51)	0.78(0.61,0.95)
75+	Both	26526.20(23024.46,29949.12)	141.76(123.05,160.05)	168546.01(137581.81,196316.00)	255.34(208.43,297.41)	535.39(398.09,692.81)	2.15(2.04,2.25)
	Male	12894.20(10626.34,15251.78)	170.23(140.29,201.35)	100158.09(78593.14,125435.59)	349.13(273.96,437.24)	676.77(455.52,939.42)	2.66(2.53,2.79)
	FemAle	13632.00(11078.01,16390.47)	122.40(99.47,147.17)	68387.92(51042.11,87168.09)	183.25(136.77,233.57)	401.67(260.37,588.96)	1.41(1.32,1.50)

ASRs, age-standardized rates (ASRs); EAPC, estimated annual percentage change; UI, uncertainty interval; CI, confidence interval.

**Figure 2:**
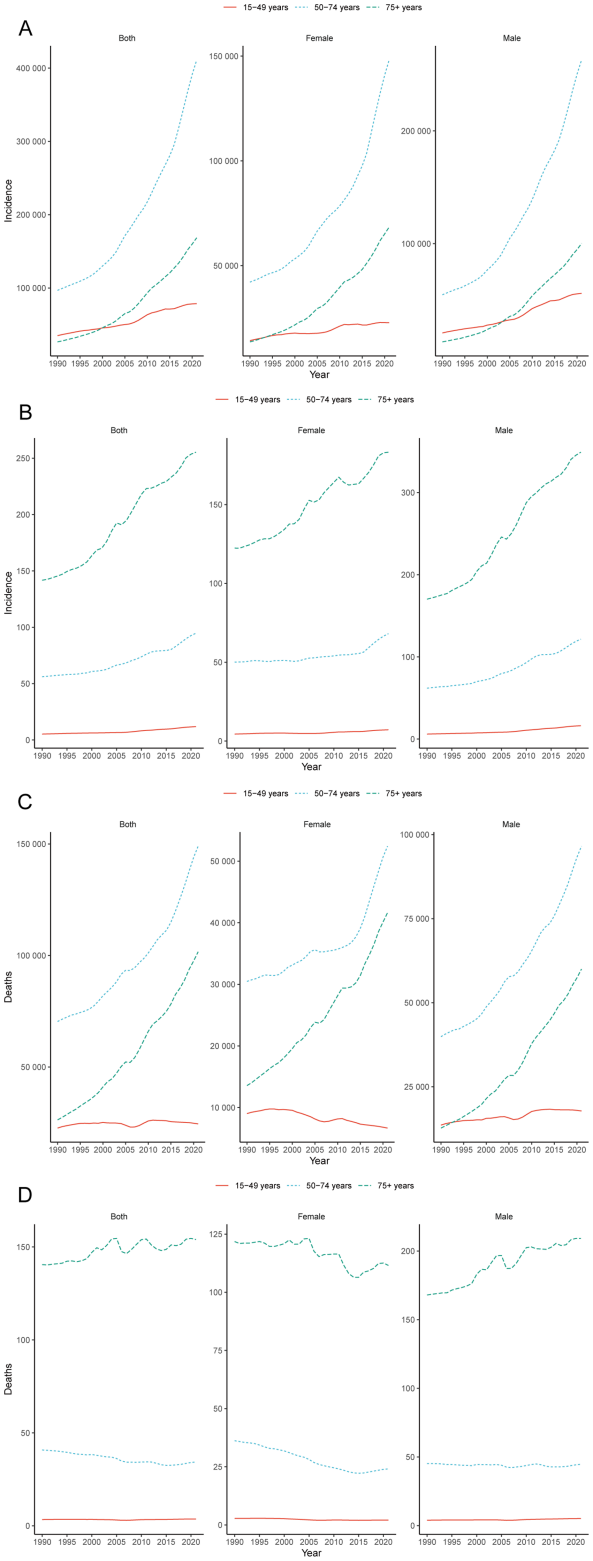
Global trends. Trends in cases **(A)** and rates **(B)** of incidence in females and males aged 15–49, 50–74 and 75+ year groups from 1990 to 2021; trends in cases **(C)** and rates **(D)** of deaths in females and males aged 15–49, 50–74 and 75 + year groups from 1990 to 2021.

In China, the age group that emerged as having the highest number of CRC cases was 50–74 years. In 2021, the number of cases in this age group was 411083.08 (95% UI: 327662.95, 500243.61), with 262928.84 (95% UI: 197241.57, 344477.29) new cases in males (Figure 2A and Table 1). In 2021, the age group with the highest incidence rate of CRC was 75 + years, with 255.34 cases per 100,000 people (95% UI: 208.43, 297.41). In males, the incidence rate was particularly high, at 349.13 cases per 100,000 people (95% UI: 273.96, 437.24) (Figure 2B and Table 1). However, it is worth noting that from 1990 to 2021, the age group with the highest EAPC increase was 15–49 years, with an EAPC increase of 2.67% (95% CI: 2.43, 2.91). The EAPC increase in the incidence rate in males was 3.40% (95% CI: 3.17, 3.63) (Figure 2B and Table 1). The trends in the prevalence and incidence of CRC in China were consistent, with the highest number of prevalent cases in the 50–74 years age group and the highest prevalence rate in the 75 + years age group (Supplementary Figures 1A,B and Supplementary Table 1). The number of deaths due to CRC increased significantly in all the male age groups, especially in the 75 + years age group. From 1990 to 2021, the number of deaths due to CRC in males increased by 371.70% (95% UI: 238.94, 524.10) (Figure 2C and Supplementary Table 2). During the period from 1990 to 2021, among different age groups, only the mortality rate due to CRC in the 75 + year age group increased, with an overall EAPC increase of 0.30% (95% CI: 0.22, 0.37) and an EAPC increase of 0.79% (95% CI: 0.71, 0.87) in males (Figure 2D and Supplementary Table 2). The mortality rate due to CRC decreased in all female age groups, with the most significant decrease in the 50–74 age group, where the EAPC decreased by 1.75% (95% CI: −1.94, −1.57). In China, the trends in DALYs and deaths due to CRC were consistent. From 1990 to 2021, DALYs increased in all the male age groups, especially in the 75+ age group, where the number of DALYs increased by 351.50% (95% CI: 221.33, 500.93) and the EAPC increased by 0.69% (95% CI: 0.59, 0.78) (Supplementary Figures 1C,D and Supplementary Table 3).

### Modifiable dietary risks in CRC

**Figure 3:**
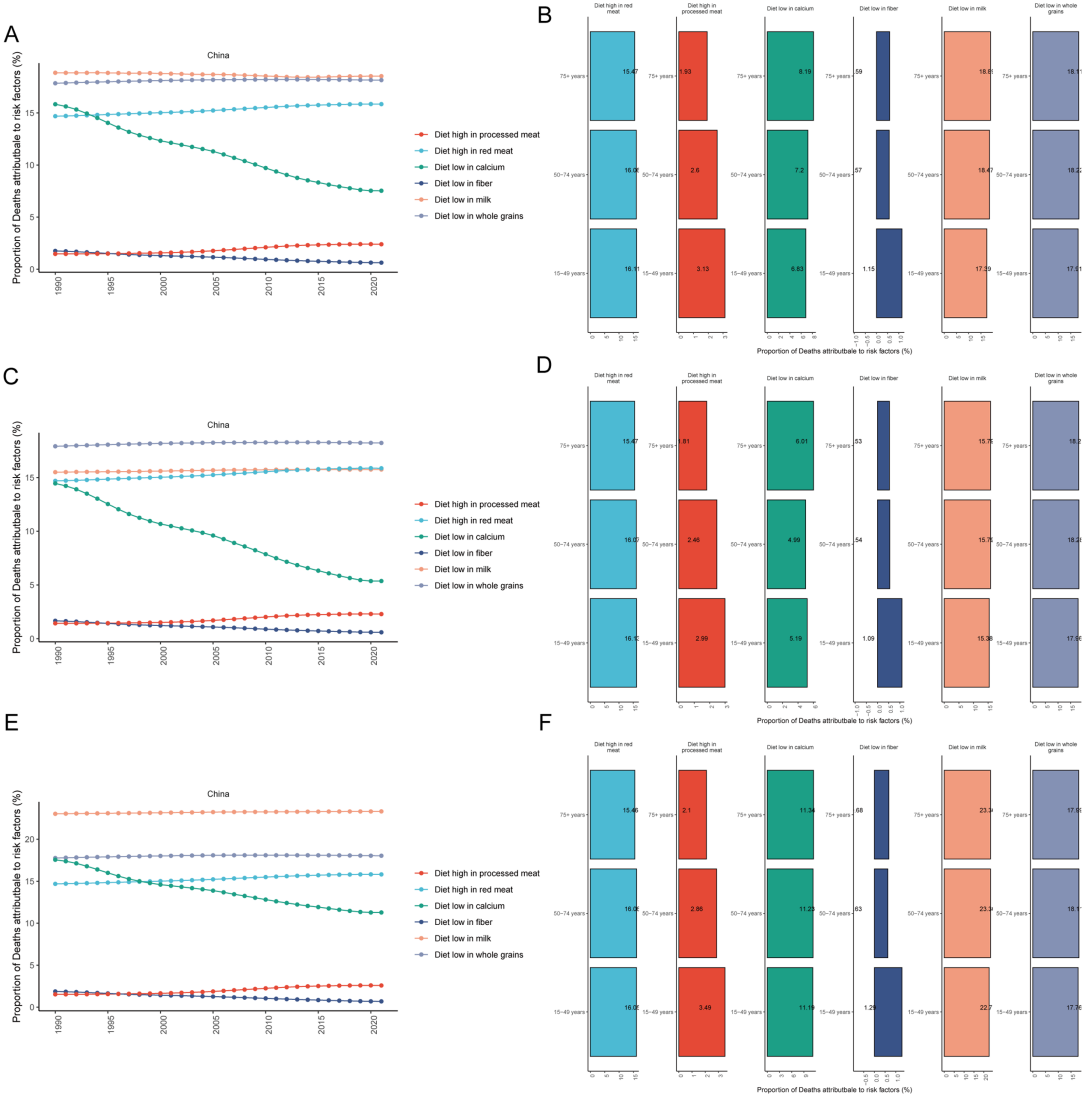
Proportional analysis of deaths attributable to diet-related risk factors for CRC. Proportional temporal trends in both **(A)**, male **(C)** and female **(E)** CRC mortality attributable to diet-related risk factors from 1990 to 2021; proportional analysis of CRC mortality attributable to diet-related risk factors in 2021 in both **(B)**, male **(D)** and female **(F)** aged 15–49, 50–74 and 75+ year groups. CRC, colorectal cancer.

From 1990 to 2021, there were six dietary risks associated with CRC mortality in both females and males: a diet high in red meat, a diet high in processed meat, a diet low in calcium, a diet low in fiber, a diet low in milk, and a diet low in whole grains (Figure 3A). Among these, the proportion of CRC deaths attributable to diets low in milk remained the highest in both females and males from 1990 to 2021. The proportion of CRC deaths attributable to a low-calcium diet decreased the most significantly (Figure 3A). In 2021, the proportion of CRC deaths attributable to diets high in processed meat was related to age, with a greater proportion in younger age groups (Figure 3B).

From 1990 to 2021, the proportion of CRC deaths attributable to diets low in whole grains was the highest in males (Figure 3C). However, the proportion of CRC deaths attributable to a low-calcium diet decreased the fastest in males (Figure 3C). In 2021, the proportion of CRC deaths in males that could be attributed to a diet high in processed meat was found to be related to age, with a greater percentage observed in younger age groups (Figure 3D).

From 1990 to 2021, the proportion of CRC deaths attributable to a diet low in milk was the highest in females (Figure 3E). Similarly, the proportion of CRC deaths attributable to a low-calcium diet decreased the fastest in females (Figure 3E). In 2021, the proportion of CRC deaths attributable to a diet high in processed meat in females was related to age, with a greater proportion in younger age groups (Figure 3F).

### Age-specific variations in diet-related CRC burden

#### Diets high in red meat

**Figure 4:**
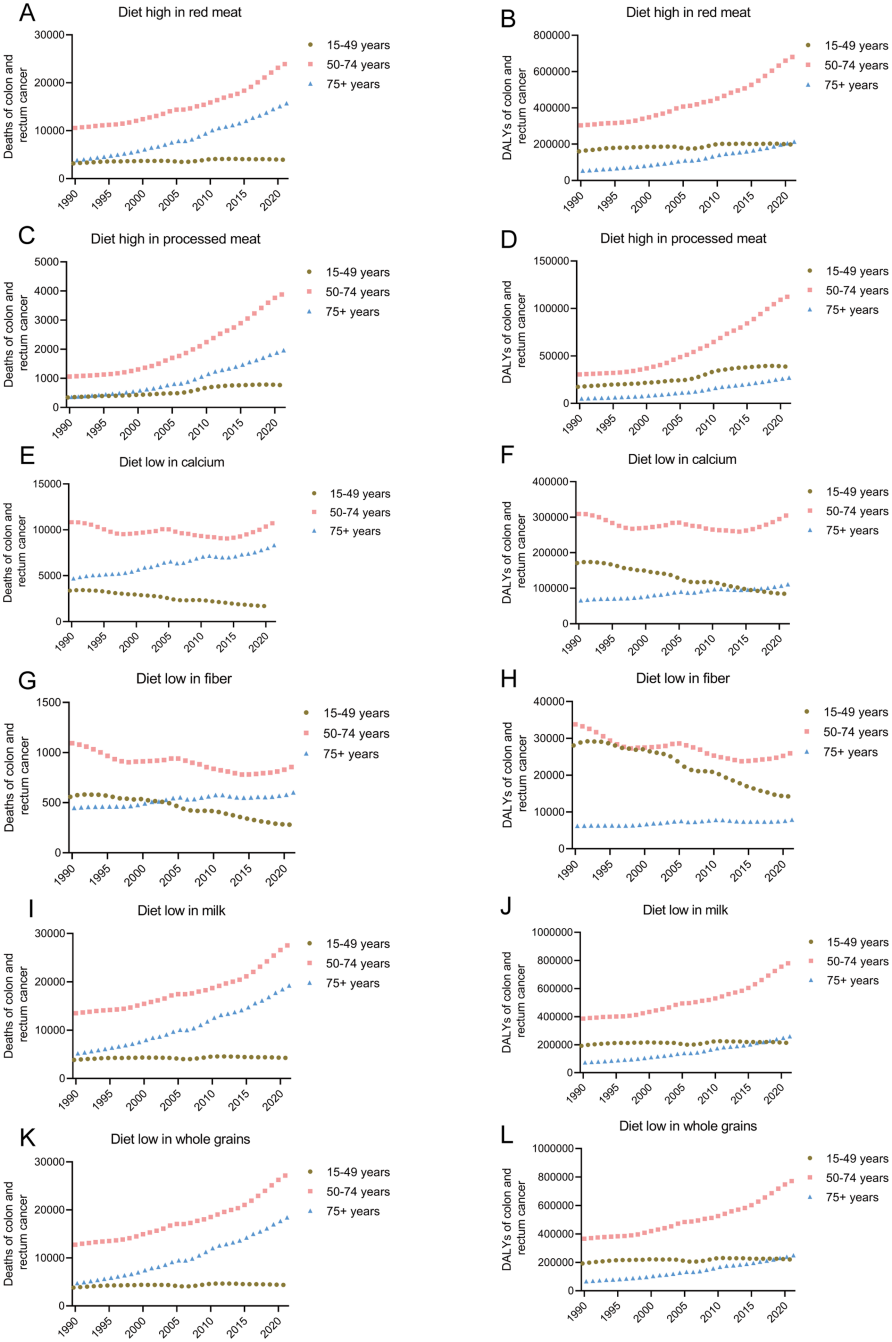
Temporal trends in mortality **(A,C,E,G,I,K)** and DALY **(B,D,F,H,J,L)** of CRC attributable to high red and processed meat, low calcium, fiber, milk and whole grains from 1990–2021. CRC, colorectal cancer; DALY, disability-adjusted life years.

In 2021, a total of 43579.90 people (95% UI: −16.08, 92082.66) died of CRC related to a diet high in red meat. From 1990 to 2021, the number of deaths and DALYs due to a diet high in red meat increased by 147.50% (95% UI: 87.98, 1911.98) and 110.68% (95% UI: 58.75, 1548.34), respectively, with EAPCs of 2.37% (95% UI: 2.27, 2.47) and 1.83% (95% UI: 1.73, 1.94). Among these, from 1990 to 2021, the number of deaths and DALYs due to CRC related to a red meat diet was the highest in the 50–74 years age group and increased the most in the 75 + years age group (Figures 4A,B and Table 2).

**Table 2:** Deaths and DALYs of colorectal cancer attributable to diet-related risk factors both male and female in China from 1990–2021.

Metric	Risk factors	1990		2021		Case percent change (%) (95% UI)	EAPC (%) (95% CI)
		Case	ASRs	Case	ASRs		
Deaths	Diet low in milk	22540.77(5848.69,37330.92)	1.92(0.50,3.17)	51030.32(13916.19,86781.25)	3.59(0.98,6.10)	126.39(73.79,190.89)	2.00(1.90,2.11)
	Diet low in whole grains	21329.92(8463.32,32783.64)	1.81(0.72,2.79)	49990.79(20099.59,79928.55)	3.51(1.41,5.62)	134.37(76.41,200.60)	2.14(2.05,2.24)
	Diet low in fiber	2100.37(927.20,3451.54)	0.18(0.08,0.29)	1738.80(693.49,3023.70)	0.12(0.05,0.21)	−17.21(−46.13,21.28)	−1.35(−1.46,−1.25)
	Diet high in red meat	17607.87(−3.39,36613.37)	1.50(−0.00,3.11)	43579.90(−16.08,92082.66)	3.06(−0.00,6.47)	147.50(87.98,1911.98)	2.37(2.27,2.47)
	Diet low in calcium	18902.66(13485.96,24551.48)	1.61(1.15,2.09)	20719.00(14553.02,28271.97)	1.46(1.02,1.99)	9.61(−17.03,42.08)	−0.48(−0.60,−0.35)
	Diet high in processed meat	1764.60(−413.69,3790.06)	0.15(−0.04,0.32)	6612.11(−1355.06,14767.43)	0.46(−0.10,1.04)	274.71(187.52,394.04)	4.17(3.95,4.39)
DALYs	Diet low in milk	652029.04(167977.40,1085153.89)	55.42(14.28,92.24)	1253643.02(337481.84,2128072.72)	88.11(23.72,149.58)	92.27(46.15,150.93)	1.47(1.36,1.57)
	Diet low in whole grains	624948.36(248199.78,959563.32)	53.12(21.10,81.56)	1241927.75(503164.76,1978508.33)	87.29(35.37,139.06)	98.72(48.92,157.14)	1.59(1.50,1.69)
	Diet low in fiber	68081.50(29789.69,111751.10)	5.79(2.53,9.50)	48099.59(18866.72,84760.77)	3.38(1.33,5.96)	−29.35(−54.34,7.60)	−1.89(−2.00,−1.79)
	Diet high in red meat	518212.88(−104.50,1074174.40)	44.05(−0.01,91.31)	1091787.60(−508.62,2295778.88)	76.74(−0.04,161.36)	110.68(58.75,1548.34)	1.83(1.73,1.94)
	Diet low in calcium	546368.99(389713.79,718067.19)	46.44(33.13,61.04)	500468.35(356218.76,682873.06)	35.18(25.04,48.00)	−8.40(−31.60,22.46)	−1.06(−1.18,−0.94)
	Diet high in processed meat	53167.50(−12352.58,115432.45)	4.52(−1.05,9.81)	178186.62(−37002.76,405024.52)	12.52(−2.60,28.47)	235.14(155.92,346.50)	3.84(3.60,4.08)

ASRs, age-standardized rates (ASRs); EAPC, estimated annual percentage change; UI, uncertainty interval; CI, confidence interval; DALY, disability-adjusted life years.

#### Diets high in processed meat

In 2021, a total of 6612.11 people (95% UI: −1355.06, 14767.43) died of CRC related to a diet high in processed meat. From 1990 to 2021, the number of deaths and DALYs due to diets high in processed meat increased by 274.71% (95% UI: 187.52, 394.04) and 235.14% (95% UI: 155.92, 346.50), with EAPCs increasing by 4.17% (95% UI: 3.95, 4.39) and 3.84% (95% UI: 3.60, 4.08), respectively. Among these, from 1990 to 2021, the number of deaths and DALYs due to CRC related to a diet high in processed meat was the highest in the 50–74 age group. The number of deaths and DALYs increased the most in the 75+ years age group, with increases of 446.39% (95% UI: 320.21, 607.87) and 271.46% (95% UI: 190.25, 364.41), respectively (Figures 4C,D and Table 2).

#### Diets low in calcium

In 2021, a total of 20719.00 people (95% UI: 14553.02, 28271.97) died of CRC related to a diet low in calcium. From 1990 to 2021, the number of deaths due to a diet low in calcium did not change significantly, with a decreasing trend in the mortality rate and an EAPC of −0.48% (95% UI: −0.60, −0.35). Among these, from 1990 to 2021, the number of deaths and DALYs due to CRC related to a diet low in calcium was the highest in the 50–74 age group. The number of deaths and DALYs increased the most in the 75+ age group, with increases of 76.94% (95% UI: 37.58, 126.26) and 26.91% (95% UI: −34.09, 127.00), respectively. In the 15–49 year age group, the number of deaths and DALYs due to a low-calcium diet decreased the most, with decreases of 50.33% (95% CI: −63.72, −32.36) and 50.47% (95% CI: −63.57, −32.84), and the EAPC decreased by 2.57% (95% CI: −2.71, −2.42) and 2.61% (95% CI: −2.77, −2.45), respectively (Figures 4E,F and Table 2).

#### Fiber-poor diets

In 2021, a total of 1738.80 people (95% UI: 693.49, 3023.70) died of CRC related to a diet low in fiber. From 1990 to 2021, the number of deaths due to a low-fiber diet decreased by 17.21% (95% UI: −46.13, 21.28), with a decreasing trend in the mortality rate and an EAPC of −1.35% (95% UI: −1.46, −1.25). Among these, from 1990 to 2021, the number of deaths due to CRC related to a low-fiber diet was the highest in the 50–74 year age group, and the mortality rate decreased the most, with an EAPC decrease of −3.95% (95% UI: −4.07, −3.84) (Figures 4G,H and Table 2).

#### Diets low in milk

The disease burden of CRC related to a diet low in milk was the heaviest among the six risk factors. In 2021, a total of 51030.32 people (95% UI: 13916.19, 86781.25) died of CRC related to a diet low in milk. From 1990 to 2021, the number of deaths and DALYs due to low milk diets increased by 126.39% (95% UI: 73.79, 190.89) and 92.27% (95% UI: 46.15, 150.93), with EAPCs increasing by 2.00% (95% CI: 1.90, 2.11) and 1.47% (95% CI: 1.36, 1.57), respectively. Among these, from 1990 to 2021, the number of deaths and DALYs due to CRC related to a diet low in milk was the highest in the 50–74 year age group. The number of deaths and DALYs increased the most in the 75+ year age group, with increases of 269.60% (95% UI: 192.51, 360.38) and 253.71% (95% UI: 177.03, 343.14), respectively (Figures 4I,J and Table 2).

#### Diets low in whole grains

In 2021, a total of 49990.79 people (95% UI: 20099.59, 79928.55) died of CRC related to a diet low in whole grains. From 1990 to 2021, the number of deaths and DALYs due to diets low in whole grains increased by 134.37% (95% UI: 76.41, 200.60) and 235.14% (95% UI: 155.92, 346.50), with EAPCs increasing by 2.14% (95% UI: 2.05, 2.24) and 3.84% (95% UI: 3.60, 4.08), respectively. Among these, from 1990 to 2021, the number of deaths and DALYs due to CRC related to a diet low in whole grains was the highest in the 50–74 age group. The number of deaths and DALYs increased the most in the 75+ year age group, with increases of 287.48% (95% UI: 205.16, 377.60) and 68.42% (95% UI: 29.87, 117.61), respectively (Figures 4K,L and Table 2).

## Discussion

The CRC ranks among the top three malignancies globally, posing a significant public health challenge in China and worldwide (15). In 2019, China reported age-standardized CRC incidence rates and mortality rates of 30.55 and 13.86 per 100,000 population, respectively (16), reflecting a substantial national burden. Dietary factors are critical contributors to CRC pathogenesis. In 2019, diet-attributable CRC mortality in China reached 90.41 per 1,000 people (9). This study systematically analyzes China’s overall CRC burden and evaluates the impact of seven dietary risk factors via data from the GBD 2021 study.

A substantial increase in the burden of colorectal cancer is evident in China. Our findings revealed 658,321 new CRC cases and 275,129 CRC-related deaths in 2021, indicating significant increases since 1990 and aligning with trends reported by Li et al. (16). The increasing incidence is multifactorial and driven by China’s economic development, shifts in living standards, and lifestyle changes. Early-onset CRC is associated with more than a dozen risk factors, including sex, ethnicity, family history, obesity, hypertension, metabolic syndrome, smoking, alcohol consumption, sedentary lifestyles, red meat intake, processed meat consumption, and sugar-sweetened beverages—all closely linked to unhealthy lifestyle practices (4, 5). Individuals with high physical activity levels (≥8,000 met-minutes/week) are at significantly lower risk of colon cancer than those with low activity levels (<600 met-minutes/week) (17). Concurrently, improved detection rates through colonoscopy screening, driven by economic and cultural advancements (18), may partially explain the increasing incidence of CRC.

Dietary factors represent a key contributor to the increasing burden of CRC, with notable disparities observed across age groups and between sexes. The increasing incidence of early-onset CRC is correlated with genetic alterations (19). A recent case–control study (7,903 CRC patients; 30,418 controls) revealed that antibiotic-exposed participants had a nearly 50% greater risk of early-onset colorectal cancer (8). Genetic predisposition also contributes to early-onset CRC, as pathogenic germline variants (20) and KRAS or SMAD4 mutations are common in younger patients (19). Notably, younger CRC patients have more immunosuppressive tumor microenvironments than older patients (21). Accumulating evidence links environmental chemical exposure during early life periods to increased colorectal cancer risk (6). Chinese birth cohorts from the 1950s–1960s (chemical introduction era) presented an increased incidence of colorectal cancer (6). Gender disparities are evident, with males demonstrating heightened susceptibility to diet-associated CRC. Males face greater colorectal cancer mortality than females from red/processed meat consumption (22), potentially due to androgen-driven increases in tumorigenic gut pathogens (23). These findings collectively highlight the serious public health challenge posed by diet as a modifiable risk factor for early-onset colorectal cancer. Multifaceted approaches targeting modifiable CRC risks—such as antibiotic stewardship, genetic screening, environmental regulation, and sex-specific dietary guidance—could mitigate this emerging trend in younger populations.

The burden of CRC attributable to diet, a modifiable risk factor, is persistently increasing. China’s urbanization and economic development accelerate a nutrition transition from traditional diets to western patterns featuring elevated processed meat and diminished whole grain/dairy consumption—which parallels in developing economies worldwide (24). This study and prior research (25) confirm that red and processed meats are critical dietary risk factors. For example, nitrite concentrations in processed meats are directly associated with an increased risk of colorectal cancer incidence (26). Red and processed meat may also increase CRC risk through interactions with genetic loci (27). The iron abundant in red meat reactivates dormant telomerase subunits via iron-sensing pirin protein, bypassing tumorigenic rate-limiting steps (28), whereas its Neu5Gc glycan promotes colorectal carcinogenesis through Wnt pathway upregulation (29). Low milk consumption significantly increases the CRC burden (30); increasing daily intake by 200 mL reduces risk (31) through milk-derived micronutrients that combat carcinogenesis (32) and microbiota-modulating components that inhibit tumor development (33). High fiber content reduces CRC risk by increasing fecal bulk, accelerating intestinal transit, and promoting short-chain fatty acid production through microbial fermentation (34). Fiber components synergize with butyrate to inhibit colon cancer cell growth and induce apoptosis (35). Interestingly, low calcium intake was associated with reduced CRC mortality in this study, which aligns with Ti et al.’s (36) findings. This study revealed that a decrease in the calcium-attributable CRC burden is correlated with improved dietary calcium intake in China, where drinking water supplies 51.59% of the daily requirements in certain populations (37). In summary, the CRC burden is driven by multiple dietary factors, including excessive consumption of red meat and processed meats alongside inadequate intake of milk, calcium, fiber, and whole grains. Addressing these modifiable dietary risks through evidence-based nutritional interventions represents a critical strategy for CRC prevention and burden reduction.

Preventive Strategies. China’s CRC burden continues to increase, with unhealthy diets contributing significantly. Reducing this burden remains a long-term public health priority. First, Chinese populations should implement dietary modifications to prevent colorectal cancer. Healthier dietary patterns—characterized by reduced red/processed meat and increased whole grains, dairy (including calcium-rich options), and fiber—should be incorporated into food production standards (38). Chinese regulatory authorities must develop frameworks that incentivize manufacturers to prioritize such health-oriented products. Second, addressing the alarming rise of early-onset diet-associated colorectal cancer requires China’s education authorities to intensify school-based health promotion (39) and enforce cafeteria compliance with national dietary guidelines to ensure student access to nutritious meals. Third, reducing exposure to controllable chemical hazards—particularly nitrosamines in processed meats (40)—while increasing the levels of protective components such as vitamin D (41), probiotics (42, 43), and omega-3 fatty acids (44)—have been shown to reduce the risk of colorectal cancer. Finally, to address the increasing trend of early-onset CRC, targeted screening for high-risk groups is essential (45). Chinese health authorities should establish comprehensive strategies (45)—including insurance reimbursement schemes—to promote early screening accessibility.

### Strengths and limitations of the study

Despite its comprehensiveness, GBD data carry inherent methodological limitations. First, GBD 2021 dietary exposure data are derived primarily from national nutrition surveys and data from the Food and Agriculture Organization of the United Nations, with residual measurement errors persisting despite calibration. Although GBD employs spatiotemporal Gaussian process regression to reduce ecological bias, population-level exposure estimates obscure individual risk profiles and preclude causal diet-CRC inferences; therefore, we interpret findings as associations rather than causal effects (14). Furthermore, the lack of publicly available provincial-level data from the GBD 2021 prevents in-depth analysis of regional dietary patterns and economic determinants across China. Third, uncontrolled confounding might persist, potentially from factors such as genetic susceptibility, physical activity, and alcohol intake. Despite its limitations, the GBD dataset remains a highly authoritative and widely recognized source in public health and disease burden research. Consequently, our findings still offer valuable insights for China to devise targeted prevention and treatment strategies.

## Conclusion

China’s CRC burden increased surged dramatically from 1990 to 2021, with key modifiable dietary risks showing population-specific impacts: low whole-grain intake disproportionately contributes to male mortality, while inadequate milk consumption is associated with increased female fatalities, and processed meat exposure is correlated with increased early-onset CRC burden. Therefore, future precision interventions and rigorously designed interventional studies are essential to validate evidence-based dietary recommendations. Concurrently, China’s National Health Commission should develop precision dietary prevention strategies informed by these findings—implementing youth-focused school nutrition programs, gender-specific supplementation initiatives, and expanded sub-50 screening coverage to mitigate this escalating burden.

## Data Availability

The original contributions presented in the study are included in the article/Supplementary material, further inquiries can be directed to the corresponding author.

## Footnotes

^1^ https://vizhub.healthdata.org/gbd-results
