# Supplementary material for: China’s colorectal cancer burden and dietary risk factors: a temporal analysis (1990–2021)
Source: Front Nutr. 2025 Jul 18;12:1590117. doi: 10.3389/fnut.2025.1590117 (PMC12313469; doi:10.3389/fnut.2025.1590117)
Supplement: Supplementary file 1 [file Image_1.pdf]

Supplementary Figures

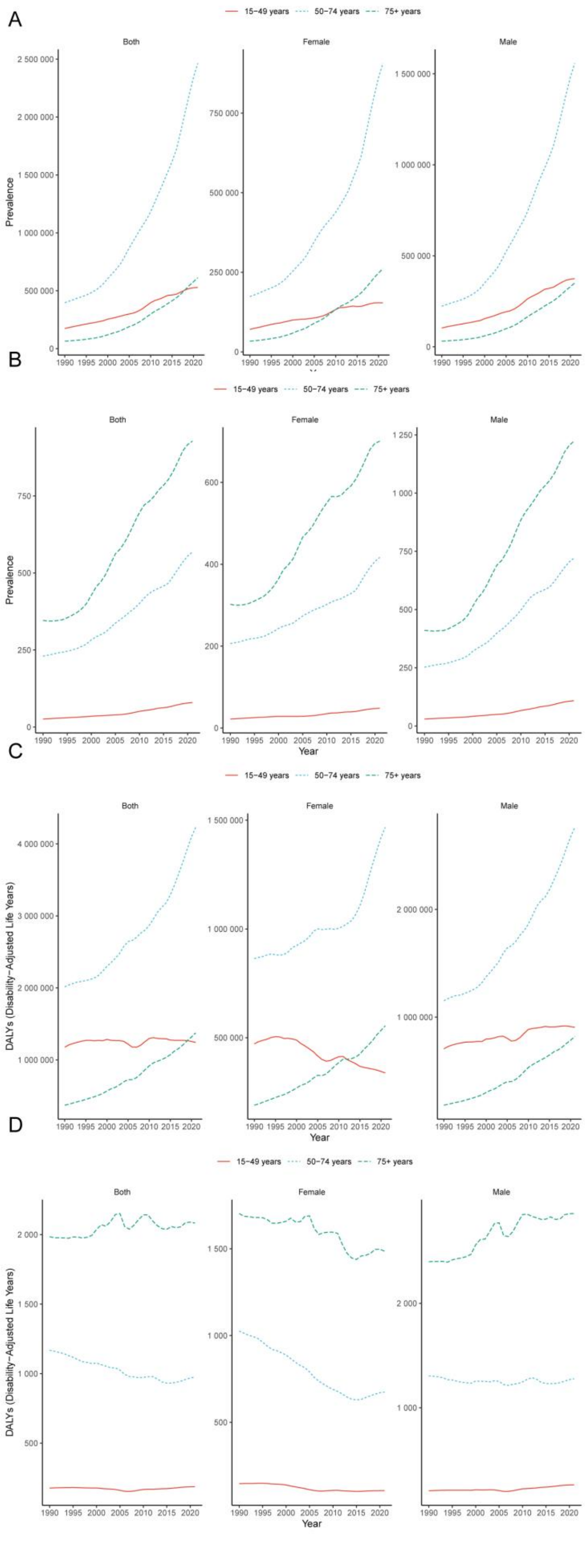

Supplementary Figure 1. Global trends. Trends in the number (A) and rate (B) of prevalence in

female and male aged 15-49, 50-74 and 75+ year group from 1990 to 2021; Trends in the number (C) and rate (D) of DALYs in female and male aged 15-49, 50-74 and 75+ year group from 1990 to 2021. CRC, colorectal cancer.
