# Supplementary material for: China’s colorectal cancer burden and dietary risk factors: a temporal analysis (1990–2021)
Source: Front Nutr. 2025 Jul 18;12:1590117. doi: 10.3389/fnut.2025.1590117 (PMC12313469; doi:10.3389/fnut.2025.1590117)
Supplement: Supplementary file 2 [file Table_1.pdf]

Supplementary Table 1. Prevalence of colorectal cancer in China between 1990 and 2021

Age(y)	Gender	1990 (95% UI)		2021 (95% UI)		Case percent change (%) (95% UI)	EAPC (%) (95% CI)
		case	ASRs	case	ASRs		
all	both	635609.34(548090.05,729557.10)	54.03(46.59,62.01)	3605686.39(2912080.79,4349689.06)	253.43(204.68,305.72)	467.28(334.76,627.13)	5.46(5.29,5.63)
	male	357597.67(286534.61,430342.62)	58.93(47.22,70.92)	2283712.16(1753654.99,2945053.95)	313.65(240.85,404.48)	538.63(347.91,802.48)	6.05(5.84,6.26)
	female	278011.67(224598.93,338303.96)	48.81(39.43,59.39)	1321974.23(1018073.47,1687270.66)	190.31(146.56,242.90)	375.51(240.05,568.92)	4.60(4.44,4.75)
15-49	both	173921.65(146987.67,201018.57)	26.08(22.04,30.14)	528566.46(420821.40,645824.11)	79.69(63.44,97.37)	203.91(128.38,300.91)	3.76(3.57,3.95)
	male	103327.73(78486.62,124956.18)	29.98(22.77,36.26)	374713.83(281555.23,486260.66)	108.74(81.70,141.11)	262.65(155.55,410.14)	4.48(4.29,4.68)
	female	70593.91(55406.56,88690.46)	21.91(17.20,27.53)	153852.63(112853.52,204375.64)	48.28(35.41,64.13)	117.94(47.25,225.29)	2.45(2.28,2.63)
50-74	both	396909.90(340048.50,458336.63)	230.07(197.11,265.68)	2464678.05(1968606.32,2979845.98)	568.15(453.80,686.91)	520.97(375.93,707.25)	3.15(3.03,3.27)
	male	223148.89(178300.47,270553.23)	252.66(201.88,306.33)	1558088.88(1177504.55,2039722.83)	720.52(544.52,943.24)	598.23(383.61,900.87)	3.79(3.63,3.95)
	female	173761.01(139682.31,211812.15)	206.38(165.90,251.57)	906589.17(691813.48,1166193.08)	416.71(317.99,536.03)	421.74(271.24,630.49)	2.24(2.12,2.36)
75+	both	64777.79(56258.33,74357.37)	346.18(300.65,397.38)	612441.87(509329.59,709364.09)	927.83(771.62,1074.67)	845.45(644.74,1092.56)	3.80(3.60,4.00)
	male	31121.05(26303.85,36762.41)	410.85(347.26,485.33)	350909.45(282190.58,433410.86)	1223.19(983.65,1510.77)	1027.56(731.09,1393.73)	4.26(4.02,4.49)

	female	33656.74(28075.53,39631.97)	302.20(252.09,355.85)	261532.42(201603.28,329466.71)	700.79(540.21,882.83)	677.06(472.21,940.90)	3.18(3.00,3.35)

ASRs, age-standardized rates (ASRs); EAPC, estimated annual percentage change; UI,uncertainty interval; CI,confidence interval

Supplementary Table 2. Deaths of colorectal cancer in China between 1990 and 2021

Age(y)	Gender	1990 (95% UI)		2021 (95% UI)		Case percent change (%) (95% UI)	EAPC (%) (95% CI)
		case	rate	case	rate		
all	both	119303.50(102706.42,137153.13)	10.14(8.73,11.66)	275129.23(223378.58,330960.39)	19.34(15.70,23.26)	130.61(73.52,195.56)	2.09(1.99,2.19)
	male	66235.44(52778.52,80322.11)	10.91(8.70,13.24)	174399.89(133841.76,226279.73)	23.95(18.38,31.08)	163.30(82.22,275.28)	2.70(2.61,2.79)
	female	53068.06(42217.24,64519.10)	9.32(7.41,11.33)	100729.34(76598.24,128091.27)	14.50(11.03,18.44)	130.61(73.52,195.56)	1.20(1.03,1.36)
15-49	both	22619.46(18985.71,26252.24)	3.39(2.85,3.94)	24475.98(19469.18,29983.10)	3.69(2.94,4.52)	8.21(-19.73,43.76)	0.08(-0.12,0.28)
	male	13597.44(10319.23,16609.72)	3.95(2.99,4.82)	17808.58(13403.39,23235.48)	5.17(3.89,6.74)	30.97(-9.26,88.21)	0.84(0.66,1.01)
	female	9022.02(6987.69,11474.79)	2.80(2.17,3.56)	6667.39(4831.89,8938.14)	2.09(1.52,2.80)	-26.10(-50.50,11.41)	-1.37(-1.61,-1.14)
50-74	both	70394.44(60008.25,81403.44)	40.80(34.78,47.19)	149006.01(118301.92,181693.18)	34.35(27.27,41.88)	111.67(57.73,178.61)	-0.76(-0.86,-0.67)
	male	39913.97(31919.22,49008.71)	45.19(36.14,55.49)	96572.51(72402.10,127452.63)	44.66(33.48,58.94)	141.95(63.43,252.06)	-0.11(-0.17,-0.04)
	female	30480.47(24343.49,3	36.20(28.91,44.57	52433.51(40073.29,67269.	24.10(18.42,30.9	72.02(20.70,144.06)	-1.75(-1.94,-1.57)

		7528.94)	)	20)	2)		
75+	both	26289.60(22841.79,29626.57)	140.50(122.07,158.33)	101647.24(83033.98,118760.87)	153.99(125.79,179.92)	286.64(205.21,381.17)	0.30(0.22,0.37)
	male	12724.03(10587.69,15039.20)	167.98(139.78,198.54)	60018.80(47061.05,75258.51)	209.21(164.04,262.33)	371.70(238.94,524.10)	0.79(0.71,0.87)
	female	13565.58(10984.20,16348.74)	121.80(98.63,146.79)	41628.44(31462.94,52730.02)	111.55(84.31,141.29)	206.87(122.89,324.63)	-0.41(-0.51,-0.31)

ASRs, age-standardized rates (ASRs); EAPC, estimated annual percentage change; UI,uncertainty interval; CI,confidence interval

Supplementary Table 3. DALY of colorectal cancer in China between 1990 and 2021

Age(y)	Gender	1990 (95% UI)		2021 (95% UI)		Case percent change (%) (95% UI)	EAPC (%) (95% CI)
		case	ASRs	case	ASRs		
all	both	3565195.70(3027610.19,4106701.24)	303.05(257.35,349.07)	6848389.89(5513406.57,8284228.27)	481.35(387.52,582.27)	92.09(43.73,149.56)	1.48(1.37,1.59)
	male	2039861.32(1602505.25,2481119.67)	336.15(264.07,408.86)	4488271.49(3427061.80,5852470.92)	616.43(470.68,803.80)	120.03(51.11,218.08)	2.10(2.00,2.21)
	female	1525334.38(1200992.93,1884417.60)	267.78(210.84,330.82)	2360118.41(1798151.90,3027362.66)	339.76(258.86,435.82)	54.73(8.30,122.08)	0.51(0.34,0.67)
15-49	both	1177805.62(988155.01,1365275.58)	176.62(148.18,204.73)	1243552.26(983058.92,1524383.13)	187.48(148.21,229.82)	5.58(-21.70,39.98)	-0.03(-0.24,0.18)
	male	705876.97(529354.09,858436.31)	204.81(153.60,249.08)	905209.32(682207.06,1174516.85)	262.68(197.97,340.83)	28.24(-10.50,83.38)	0.73(0.55,0.91)
	female	471928.65(364596.07,600389.14)	146.47(113.15,186.33)	338342.95(244200.13,455143.39)	106.16(76.62,142.81)	-28.31(-51.93,8.19)	-1.48(-1.72,-1.24)
50-74	both	2016004.79(1712083.32,2344434.65)	1168.58(992.41,1358.96)	4229874.76(3353366.77,5147564.91)	975.06(773.01,1186.60)	109.81(55.66,178.20)	-0.73(-0.82,-0.65)
	male	1152278.74(917326.98,1417407.68)	1304.65(1038.63,1604.83)	1467212.34(1107532.53,1901717.80)	1277.55(955.03,1691.04)	139.76(61.71,251.58)	-0.07(-0.14,-0.01)
	female	863726.05(685962.82,1067123.79)	1025.85(814.72,1267.43)	2762662.43(2065212.73,3656821.20)	674.39(509.07,874.11)	69.87(18.41,144.64)	-1.74(-1.91,-1.57)
75+	both	371385.29(323600.25,419403.73)	1984.74(1729.37,2241.36)	1374962.87(1121843.90,1623508.63)	2083.04(1699.57,2459.58)	270.23(190.85,365.29)	0.19(0.11,0.27)
	male	181705.62(150304.59,215322.68)	2398.83(1984.28,2842.64)	820399.75(639148.60,1031789.16)	2859.72(2227.92,3596.58)	351.50(221.33,500.93)	0.69(0.59,0.78)
	female	189679.68(154652.34	1703.11(1388.61,	554563.12(419819.68,70	1485.99(1124.93	192.37(111.13,304.07)	-0.54(-0.64,-0.44)

	,227688.66)	2044.39)	4904.03)	,1888.83)

ASRs, age-standardized rates (ASRs); EAPC, estimated annual percentage change; UI,uncertainty interval; CI,confidence interval; DALY, disability-adjusted life years
